# Supplementary material for: Broad-Spectrum Salmonella Phages PSE-D1 and PST-H1 Controls Salmonella in Foods
Source: Viruses. 2022 Nov 27;14(12):2647. doi: 10.3390/v14122647 (PMC9784834; doi:10.3390/v14122647)
Supplement: Supplementary file 1 [file viruses-14-02647-s001.zip › viruses-1960237-supplementary.pdf]

Table S1. CDS function prediction of phage PSE-D1.

CDS	Hit_name	Hit_description	Length (aa)	Strand	Identity (%)	Value
1	WP_153932247.1	hypothetical protein [Klebsiella pneumoniae]	297	-	100.00%	0.00E+00
2	UNY41078.1	rIIB lysis inhibitor protein [Klebsiella phage KP182]	912	-	100.00%	1.00E-176
3	QLF83147.1	rIIA lysis inhibitor [Klebsiella phage KpnM6E1]	2169	-	99.45%	0.00E+00
4	YP_010089307.1	rIIA lysis inhibitor [Klebsiella phage KPV15]	207	-	97.06%	2.00E-25
5	QQM14529.1	hypothetical protein [Klebsiella phage vB_KpnM_17-11]	153	-	98.00%	2.00E-27
6	UJP30193.1	DNA topoisomerase large subunit [Klebsiella phage Kpn6N]	1839	-	99.67%	0.00E+00
7	UMM76775.1	hypothetical protein [Klebsiella phage UTI-K1]	369	-	96.72%	5.00E-80
8	AUV57630.1	hypothetical protein [Klebsiella phage KP1]	249	-	98.78%	3.00E-52
9	QQM14524.1	hypothetical protein [Klebsiella phage vB_KpnM_17-11]	177	-	89.66%	9.00E-31
10	QQM14523.1	hypothetical protein [Klebsiella phage vB_KpnM_17-11]	336	-	99.10%	1.00E-76
11	UMM76780.1	hypothetical protein [Klebsiella phage UTI-K1]	366	-	99.17%	1.00E-75
12	UMM77060.1	hypothetical protein [Klebsiella phage UTI-K4]	216	-	98.59%	6.00E-44
13	UMM76782.1	hypothetical protein [Klebsiella phage UTI-K1]	441	-	98.63%	3.00E-99
14	UMM77062.1	hypothetical protein [Klebsiella phage UTI-K4]	168	-	100.00%	1.00E-18
15	QPB09025.1	DexA-like exonuclease [Klebsiella phage Metamorpho]	690	-	96.07%	1.00E-144
16	UJP30204.1	hypothetical protein [Klebsiella phage Kpn6N]	255	-	97.62%	6.00E-53
17	UYE90552.1	DNA helicase [Klebsiella phage pKp20]	1332	-	99.32%	0.00E+00
18	QLF82884.1	hypothetical protein KpnM6E1_gp001 [Klebsiella phage KpnM6E1]	315	-	99.04%	2.00E-70
19	QGF21434.1	anti-termination [Klebsiella phage JIPh_Kp122]	756	-	100.00%	6.00E-173
20	YP_010089324.1	NAD--protein ADP-ribosyltransferase [Klebsiella phage KPV15]	690	-	99.56%	3.00E-170
21	UOK17627.1	molybdenum ABC transporter [Klebsiella phage	189	-	96.67%	7.00E-1

		KP1079]				0
22	UNY41100.1	hypothetical protein [Klebsiella phage KP185]	510	-	98.22%	6.00E-122
23	UWG89310.1	hypothetical protein [Bacteriophage sp.]	192	-	98.41%	7.00E-26
24	QGF21439.1	capsid and scaffold protein [Klebsiella phage JIPh_Kp122]	228	-	97.33%	5.00E-33
25	UMM76510.1	hypothetical protein [Klebsiella phage UTI-K1]	138	-	88.46%	8.00E-05
26	YP_009190590.1	dCTP pyrophosphatase [Klebsiella phage JD18]	522	-	100.00%	5.00E-125
27	YP_010089331.1	hypothetical protein KNT59_gp050 [Klebsiella phage KPV15]	192	+	98.41%	4.00E-37
28	UGO53187.1	putative DNA primase/DNA helicase [Klebsiella phage vB_KaeM_Nispero]	1023	-	99.12%	0.00E+00
29	YP_009190593.1	hypothetical protein AU097_gp012 [Klebsiella phage JD18]	165	-	97.56%	2.00E-19
30	AUV57372.1	hypothetical protein [Klebsiella phage KP1]	210	-	98.55%	6.00E-43
31	YP_010096006.1	spackle periplasmic protein [Klebsiella phage Mineola]	294	-	100.00%	4.00E-64
32	UGO49665.1	putative DNA primase/helicase [Klebsiella phage vB_KaeM_Merci]	1440	-	100.00%	0.00E+00
33	YP_010098647.1	capsid and scaffold protein [Klebsiella phage KP179]	351	-	99.14%	2.00E-76
34	UJP30223.1	recombination protein [Klebsiella phage Kpn6N]	1176	-	99.74%	0.00E+00
35	AUV57378.1	hypothetical protein [Klebsiella phage KP1]	222	-	98.63%	8.00E-46
36	QQM14496.1	glucosyltransferase [Klebsiella phage vB_KpnM_17-11]	837	-	99.28%	0.00E+00
37	QSJ03771.1	deoxycytidylate 5-hydroxymethyltransferase [Clostridium phage HZhang-2021a]	735	-	78.37%	2.00E-139
38	OHB25415.1	hypothetical protein KNT59_gp062 [Klebsiella phage KPV15]	207	-	54.35%	1.00E-09
39	UNY41120.1	hypothetical protein [Klebsiella phage KP185]	444	-	98.64%	7.00E-103
40	QEG11007.1	DNA polymerase [Klebsiella phage KMI12]	2709	-	99.78%	0.00E+00
41	YP_009190607.1	translational repressor RegA [Klebsiella phage JD18]	372	-	100.00%	2.00E-85
42	YP_009190608.1	DNA polymerase clamp loader subunit Gp62 [Klebsiella phage JD18]	567	-	100.00%	1.00E-121
43	UJP30232.1	replication factor C small subunit / DNA polymerase	963	-	99.69%	0.00E+

		clamp loader subunit [Klebsiella phage Kpn6N]				00
44	AUV57388.1	sliding clamp, DNA polymerase accessory protein [Klebsiella phage KP1]	702	-	99.57%	2.00E-1 67
45	YP_0098367 69.1	RNA polymerase [Klebsiella phage vB_Kpn_F48]	378	-	70.73%	3.00E-5 6
46	QEG11811.1	hypothetical protein KPN6_151 [Klebsiella phage KPN6]	195	-	98.44%	1.00E-3 9
47	AUV57391.1	recombination-related endonuclease [Klebsiella phage KP1]	1683	-	99.46%	0.00E+ 00
48	UNY41129.1	recombination-related endonuclease [Klebsiella phage KP185]	1023	-	92.35%	0.00E+ 00
49	QGF21466.1	alpha glucosyl transferase [Klebsiella phage JIPh_Kp122]	1197	-	99.25%	0.00E+ 00
50	UCR74262.1	hypothetical protein [Klebsiella phage vB_KpnM_5N]	258	-	96.47%	9.00E-5 5
51	UJP30240.1	hypothetical protein [Klebsiella phage Kpn6N]	198	-	96.92%	7.00E-3 9
52	UCR74264.1	hypothetical protein [Klebsiella phage vB_KpnM_5N]	303	-	97.00%	8.00E-6 5
53	YP_0100986 26.1	hypothetical protein KNU12_gp186 [Klebsiella phage KP179]	141	-	100.00%	6.00E-2 5
54	YP_0091906 21.1	RNA polymerase subunit sigma [Klebsiella phage JD18]	549	-	100.00%	1.00E-1 33
55	AUV57399.1	hypothetical protein [Klebsiella phage KP1]	246	-	98.77%	2.00E-5 2
56	AUV57400.1	hypothetical protein [Klebsiella phage KP1]	279	-	97.83%	2.00E-5 8
57	QQM14474. 1	hypothetical protein [Klebsiella phage vB_KpnM_17-11]	225	-	98.65%	6.00E-4 5
58	AUV57402.1	hypothetical protein [Klebsiella phage KP1]	285	-	98.94%	4.00E-6 1
59	UMM76829. 1	hypothetical protein [Klebsiella phage UTI-K4]	258	-	94.12%	8.00E-5 2
60	CAD524204 2.1	phage protein [Klebsiella phage vB_KpM-Wobble]	333	-	99.09%	5.00E-7 3
61	AUV57405.1	hypothetical protein [Klebsiella phage KP1]	243	-	98.75%	1.00E-5 1
62	AUV57406.1	hypothetical protein [Klebsiella phage KP1]	189	-	95.16%	9.00E-3 7
63	YP_0092894 84.1	glutaredoxin [Klebsiella phage PKO111]	303	-	100.00%	9.00E-6 6
64	AUV57408.1	hypothetical protein [Klebsiella phage KP1]	342	-	99.12%	2.00E-7 5
65	AUV57409.1	hypothetical protein [Klebsiella phage KP1]	213	-	98.57%	2.00E-4

						0
66	QQM14466.1	hypothetical protein [Klebsiella phage vB_KpnM_17-11]	117	-	96.88%	0.00E+00
67	UMM76837.1	ribonucleotide reductase of class III (anaerobic), activating protein [Klebsiella phage UTI-K4]	471	-	99.36%	0.00E+00
68	UJP30256.1	hypothetical protein [Klebsiella phage Kpn6N]	219	-	97.22%	3.00E-44
69	QEG11487.1	ribonucleotide reductase of class III (anaerobic) [Klebsiella phage KMI13]	1839	-	100.00%	0.00E+00
70	YP_009289480.1	endonuclease VII [Klebsiella phage PKO111]	474	-	99.36%	0.00E+00
71	UMM76559.1	hypothetical protein [Klebsiella phage UTI-K1]	309	-	96.08%	1.00E-66
72	YP_010089383.1	pin protease inhibitor [Klebsiella phage KPV15]	453	-	96.00%	4.00E-101
73	QYN80518.1	hypothetical protein [Kosakonia phage Kc304]	159	-	65.85%	0.00E+00
74	QGF21489.1	nucleotide reductase subunit C [Klebsiella phage JIPh_Kp122]	390	-	99.22%	0.00E+00
75	YP_010089386.1	hypothetical protein KNT59_gp105 [Klebsiella phage KPV15]	171	-	96.43%	0.00E+00
76	UJP30265.1	hypothetical protein [Klebsiella phage Kpn6N]	189	-	98.39%	0.00E+00
77	YP_010098603.1	glutaredoxin [Klebsiella phage KP179]	270	-	97.75%	0.00E+00
78	UJP30267.1	hypothetical protein [Klebsiella phage Kpn6N]	291	-	97.92%	0.00E+00
79	UMM76850.1	hypothetical protein [Klebsiella phage UTI-K4]	159	-	96.15%	0.00E+00
80	UMM76851.1	hypothetical protein [Klebsiella phage UTI-K4]	510	-	84.62%	0.00E+00
81	UJP30270.1	hypothetical protein [Klebsiella phage Kpn6N]	333	-	99.09%	1.00E+00
82	UNY41164.1	hypothetical protein [Klebsiella phage KP185]	315	-	98.08%	2.00E+00
83	UNY41165.1	thioredoxin [Klebsiella phage KP185]	237	-	98.72%	3.00E+00
84	AUV57426.1	hypothetical protein [Klebsiella phage KP1]	975	-	98.46%	4.00E+00
85	YP_010089397.1	hypothetical protein KNT59_gp116 [Klebsiella phage KPV15]	789	-	99.62%	5.00E+00
86	UMM76574.1	hypothetical protein [Klebsiella phage UTI-K1]	195	-	98.44%	3.00E-37
87	UMM76575.	hypothetical protein [Klebsiella phage UTI-K1]	165	-	98.15%	4.00E-3

	1					0
88	YP_009190656.1	hypothetical protein AU097_gp075 [Klebsiella phage JD18]	198	-	100.00%	3.00E-40
89	QYC52776.1	hypothetical protein [Klebsiella phage vB_KpnM_TU02]	321	-	99.06%	6.00E-73
90	AUV57433.1	hypothetical protein [Klebsiella phage KP1]	336	-	99.10%	3.00E-74
91	YP_009190659.1	hypothetical protein AU097_gp078 [Klebsiella phage JD18]	426	-	100.00%	6.00E-100
92	UMM76580.1	hypothetical protein [Klebsiella phage UTI-K1]	147	-	95.83%	3.00E-25
93	AUV57436.1	thioredoxin [Klebsiella phage KP1]	978	-	99.69%	0.00E+00
94	UMM76582.1	hypothetical protein [Klebsiella phage UTI-K1]	216	-	98.59%	1.00E-42
95	UMM76583.1	hypothetical protein [Klebsiella phage UTI-K1]	216	-	98.59%	1.00E-42
96	CAD2272535.1	thioredoxin, phage-associated [Klebsiella phage vB_KpnM_311F]	1011	-	99.40%	0.00E+00
97	QYC52785.1	hypothetical protein [Klebsiella phage vB_KpnM_TU02]	219	-	100.00%	6.00E-46
98	AUV57441.1	hypothetical protein [Klebsiella phage KP1]	192	-	100.00%	4.00E-37
99	UMM76587.1	hypothetical protein [Klebsiella phage UTI-K1]	222	-	98.63%	1.00E-44
100	QYC52787.1	hypothetical protein [Klebsiella phage vB_KpnM_TU02]	582	-	98.96%	7.00E-138
101	YP_009190668.1	hypothetical protein AU097_gp087 [Klebsiella phage JD18]	492	-	99.39%	1.00E-89
102	AUV57445.1	hypothetical protein [Klebsiella phage KP1]	222	-	98.63%	2.00E-44
103	AUV57446.1	pothetical protein [Klebsiella phage KP1]	192	-	98.41%	4.00E-36
104	QBA85021.1	putative zinc-finger containing protein [Klebsiella phage vB_KpnM_GF]	267	-	98.86%	3.00E-57
105	QYC52793.1	hypothetical protein [Klebsiella phage vB_KpnM_TU02]	489	-	100.00%	2.00E-102
106	QBA85022.1	hypothetical protein [Klebsiella phage vB_KpnM_GF]	387	-	99.22%	6.00E-88
107	YP_009836719.1	hypothetical protein HWB49_gp035 [Klebsiella phage vB_Kpn_F48]	213	-	100.00%	3.00E-42
108	UNY40912.1	hypothetical protein [Klebsiella phage KP182]	384	-	99.21%	2.00E-87
109	UCR74319.1	hypothetical protein [Klebsiella phage	135	-	97.44%	3.00E-0

		vB_KpnM_5N]				8
110	QEG11812.1	hypothetical protein KPN6_208 [Klebsiella phage KPN6]	195	-	100.00%	2.00E-40
111	QYC52800.1	thymidine kinase [Klebsiella phage vB_KpnM_TU02]	600	-	99.50%	2.00E-145
112	YP_010096085.1	hypothetical protein KNT92_gp110 [Klebsiella phage Mineola]	168	-	98.18%	1.00E-30
113	QBA85025.1	hypothetical protein [Klebsiella phage vB_KpnM_GF]	468	-	98.71%	1.00E-108
114	QEG11530.1	hypothetical protein KMI13_199 [Klebsiella phage KMI13]	222	-	100.00%	7.00E-47
115	QGF21531.1	hypothetical protein JIPhKp122_0099 [Klebsiella phage JIPh_Kp122]	321	-	98.11%	2.00E-69
116	QYC52805.1	hypothetical protein [Klebsiella phage vB_KpnM_TU02]	546	-	98.90%	1.00E-128
117	UWI30169.1	endoribonuclease [Bacteriophage sp.]	459	-	99.34%	1.00E-106
118	QEG11534.1	hypothetical protein KMI13_203 [Klebsiella phage KMI13]	276	-	98.90%	2.00E-59
119	QBZ70935.1	pyruvate formate-lyase [Shigella phage SSE1]	363	-	84.17%	3.00E-67
120	UNY40924.1	hypothetical protein [Klebsiella phage KP182]	345	-	95.61%	4.00E-77
121	AUV57466.1	hypothetical protein [Klebsiella phage KP1]	312	-	98.06%	4.00E-68
122	QYC52812.1	hypothetical protein [Klebsiella phage vB_KpnM_TU02]	306	-	100.00%	2.00E-68
123	QEG10893.1	hypothetical protein KMI11_122 [Klebsiella phage KMI11]	573	-	99.47%	2.00E-133
124	YP_010096096.1	hypothetical protein KNT92_gp121 [Klebsiella phage Mineola]	480	-	98.74%	7.00E-111
125	QEG11682.1	baseplate hub + tail lysozyme [Klebsiella phage KPN6]	495	-	99.39%	5.00E-101
126	YP_009288804.1	NUDIX hydrolase [Klebsiella phage vB_KpnM_KpV477]	453	-	99.32%	3.00E-107
127	UMM76903.1	hypothetical protein [Klebsiella phage UTI-K4]	237	-	98.72%	3.00E-48
128	UMM76616.1	hypothetical protein [Klebsiella phage UTI-K1]	159	-	100.00%	4.00E-16
129	QLF83001.1	hypothetical protein KpnM6E1_gp118 [Klebsiella phage KpnM6E1]	561	-	98.39%	2.00E-115
130	UJP30322.1	hypothetical protein [Klebsiella phage Kpn6N]	594	-	92.39%	3.00E-126
131	UGO53286.1	hypothetical protein NISPERO_110 [Klebsiella	228	-	97.33%	1.00E-4

		phage vB_KaeM_Nispero]				4
132	UMM76910.1	hypothetical protein [Klebsiella phage UTI-K4]	234	-	98.70%	3.00E-48
133	UMM76623.1	hypothetical protein [Klebsiella phage UTI-K1]	291	-	98.96%	1.00E-50
134	QYC52830.1	hypothetical protein [Klebsiella phage vB_KpnM_TU02]	129	-	95.24%	2.00E-19
135	UYE90674.1	hypothetical protein CPT_pKp20_142 [Klebsiella phage pKp20]	450	-	99.33%	1.00E-98
136	UMM76627.1	hypothetical protein [Klebsiella phage UTI-K1]	558	-	97.84%	9.00E-134
137	YP_010096113.1	hypothetical protein KNT92_gp273 [Klebsiella phage Mineola]	225	-	100.00%	2.00E-44
138	QQM14391.1	hypothetical protein [Klebsiella phage vB_KpnM_17-11]	231	-	98.68%	8.00E-48
139	QEG11558.1	hypothetical protein KMI13_227 [Klebsiella phage KMI13]	369	-	99.18%	3.00E-85
140	UJP30333.1	hypothetical protein [Klebsiella phage Kpn6N]	153	-	98.00%	2.00E-26
141	USL86966.1	hypothetical protein [Salmonella phage PSE-D1]	141	+	100.00%	2.00E-23
142	UMM76632.1	hypothetical protein [Klebsiella phage UTI-K1]	351	-	98.28%	1.00E-78
143	AUV57490.1	hypothetical protein [Klebsiella phage KP1]	186	-	98.36%	7.00E-37
144	QQM14386.1	hypothetical protein [Klebsiella phage vB_KpnM_17-11]	180	-	96.61%	1.00E-09
145	YP_009190717.1	hypothetical protein AU097_gp136 [Klebsiella phage JD18]	180	-	100.00%	8.00E-23
146	YP_009190718.1	hypothetical protein AU097_gp137 [Klebsiella phage JD18]	456	-	100.00%	1.00E-108
147	UCR74357.1	hypothetical protein [Klebsiella phage vB_KpnM_5N]	237	-	98.44%	4.00E-34
148	YP_009190720.1	deoxynucleoside monophosphate kinase [Klebsiella phage JD18]	738	-	100.00%	9.00E-180
149	YP_010098538.1	tail completion protein [Klebsiella phage KP179]	603	-	99.50%	4.00E-148
150	ULA52389.1	DNA end protector during packaging [Enterobacter phage vB-EclM_KMB19]	828	-	77.26%	3.00E-159
151	YP_009289410.1	head completion protein [Klebsiella phage PKO111]	450	-	99.33%	6.00E-106
152	YP_009289411.1	baseplate wedge protein 53 [Klebsiella phage PKO111]	639	+	100.00%	2.00E-155
153	AUV57501.1	baseplate hub + tail lysozyme [Klebsiella phage KP1]	1731	+	99.48%	0.00E+

						00
154	QQM14371.1	hypothetical protein [Klebsiella phage vB_KpnM_17-11]	504	+	99.40%	2.00E-107
155	QBP35495.1	phospholipase [Phage NC-G]	294	+	82.29%	2.00E-51
156	UNY41240.1	baseplate wedge subunit [Klebsiella phage KP185]	1968	+	99.69%	0.00E+00
157	AUV57505.1	baseplate wedge initiator [Klebsiella phage KP1]	3099	+	99.90%	0.00E+00
158	YP_009190730.1	baseplate wedge subunit [Klebsiella phage JD18]	1026	+	99.69%	0.00E+00
159	AUV57507.1	baseplate wedge tail fiber connector [Klebsiella phage KP1]	912	+	99.67%	0.00E+00
160	CAD5242911.1	phage baseplate wedge [Klebsiella phage vB_KoM-Flushed]	1824	+	99.84%	0.00E+00
161	YP_009289420.1	baseplate wedge subunit and tail pin [Klebsiella phage PKO111]	672	+	99.55%	2.00E-164
162	QBA85042.1	straight tail fiber [Klebsiella phage vB_KpnM_GF]	1347	+	99.11%	0.00E+00
163	QLF83037.1	neck whiskers protein [Klebsiella phage KpnM6E1]	1767	+	98.81%	0.00E+00
164	QGF21582.1	head completion, neck hetero-dimeric protein [Klebsiella phage JIPh_Kp122]	942	+	99.36%	0.00E+00
165	QFR57189.1	head completion, neck hetero-dimeric protein [Klebsiella phage AmPh_EK29]	777	+	76.36%	6.00E-144
166	QGF21584.1	proximal tail sheath stabilization protein [Klebsiella phage JIPh_Kp122]	825	+	99.64%	0.00E+00
167	UGO48513.1	terminase small subunit [Kelbsiella phage vB_KaeM_Boboto]	510	+	99.41%	3.00E-107
168	QPB08858.1	terminase large subunit [Klebsiella phage Metamorpho]	1839	+	98.20%	0.00E+00
169	QGF21587.1	tail sheath monomer [Klebsiella phage JIPh_Kp122]	1974	+	99.24%	0.00E+00
170	YP_009836926.1	tail protein [Klebsiella phage vB_Kpn_F48]	492	+	86.42%	6.00E-95
171	AUV57519.1	portal vertex of the head [Klebsiella phage KP1]	1569	+	99.81%	0.00E+00
172	CAD2273129.1	prohead core protein [Klebsiella phage vB_KpnM_311F]	219	+	100.00%	2.00E-22
173	QGF21591.1	capsid and scaffold protein [Klebsiella phage JIPh_Kp122]	426	+	100.00%	3.00E-81
174	QGT55407.1	prohead assembly (scaffolding) protein [Escherichia phage FP43]	651	+	82.08%	1.00E-119
175	AUV57523.1	prohead assembly (scaffolding) protein [Klebsiella	813	+	99.63%	2.00E-1

		phage KP1]				50
176	AUV57524.1	major capsid protein [Klebsiella phage KP1]	1560	+	100.00%	0.00E+00
177	QGF21595.1	capsid vertex [Klebsiella phage JIPh_Kp122]	1281	+	99.77%	0.00E+00
178	AUV57526.1	hypothetical protein [Klebsiella phage KP1]	549	-	99.45%	8.00E-131
179	UJP30097.1	RNA ligase [Klebsiella phage Kpn6N]	1008	-	97.91%	0.00E+00
180	UNY41265.1	hypothetical protein [Klebsiella phage KP185]	258	-	97.65%	1.00E-54
181	UMM76959.1	hypothetical protein [Klebsiella phage UTI-K4]	327	-	97.22%	8.00E-71
182	QEG11348.1	hypothetical protein KMI13_17 [Klebsiella phage KMI13]	228	-	100.00%	4.00E-46
183	UMM76677.1	capsid and scaffold protein [Klebsiella phage UTI-K1]	1125	-	96.79%	0.00E+00
184	QBA85052.1	inhibitor of prohead protease [Klebsiella phage vB_KpnM_GF]	795	-	100.00%	4.00E-177
185	UYE90726.1	DNA helicase [Klebsiella phage pKp20]	1503	+	98.60%	0.00E+00
186	YP_010077174.1	homing endonuclease [Yersinia phage PYPS2T]	681	+	69.78%	1.00E-107
187	QBA85054.1	DNA helicase [Klebsiella phage vB_KpnM_GF]	243	+	98.53%	8.00E-40
188	UKS71665.1	hypothetical protein FRZ284_00073 [Klebsiella phage vB_KpnM_FRZ284]	168	-	97.50%	9.00E-20
189	YP_009836906.1	single stranded DNA-binding protein [Klebsiella phage vB_Kpn_F48]	417	-	78.99%	2.00E-65
190	UOK17800.1	baseplate wedge subunit [Klebsiella phage KP1079]	411	-	97.79%	9.00E-91
191	YP_009836904.1	baseplate hub assembly chaperone [Klebsiella phage vB_Kpn_F48]	630	-	63.11%	1.00E-92
192	QGF21609.1	baseplate [Klebsiella phage JIPh_Kp122]	759	+	99.60%	0.00E+00
193	YP_009190764.1	baseplate hub subunit [Klebsiella phage JD18]	1143	+	100.00%	0.00E+00
194	UNY41278.1	baseplate hub [Klebsiella phage KP185]	528	+	99.43%	2.00E-122
195	CAD5243109.1	baseplate hub [Klebsiella phage vB_KpM-Wobble]	1734	+	99.65%	0.00E+00
196	QYC52894.1	baseplate tail tube cap [Klebsiella phage vB_KpnM_TU02]	1053	+	99.71%	0.00E+00
197	QOI66406.1	tail assembly protein [Erwinia phage FBB1]	927	+	71.89%	8.00E-1

						48
198	YP_010096175.1	hypothetical protein KNT92_gp211 [Klebsiella phage Mineola]	279	-	100.00%	9.00E-60
199	QLF83072.1	RNA polymerase ADP-ribosylase [Klebsiella phage KpnM6E1]	2127	-	99.86%	0.00E+00
200	UNY41284.1	hypothetical protein [Klebsiella phage KP185]	180	-	98.31%	1.00E-32
201	YP_009190772.1	DNA ligase [Klebsiella phage JD18]	1473	-	99.80%	0.00E+00
202	UJP30119.1	hypothetical protein [Klebsiella phage Kpn6N]	207	-	97.06%	9.00E-43
203	UYE90743.1	hypothetical protein CPT_pKp20_224 [Klebsiella phage pKp20]	843	-	99.64%	0.00E+00
204	UNY41013.1	hypothetical protein [Klebsiella phage KP182]	201	-	96.97%	3.00E-40
205	QEG10809.1	hypothetical protein KMI11_38 [Klebsiella phage KMI11]	171	-	98.21%	3.00E-32
206	UMM76699.1	hypothetical protein [Klebsiella phage UTI-K1]	279	-	98.91%	4.00E-59
207	AUV57554.1	hypothetical protein [Klebsiella phage KP1]	489	-	94.44%	1.00E-96
208	QYC52907.1	hypothetical protein [Klebsiella phage vB_KpnM_TU02]	360	-	99.16%	4.00E-82
209	UMM76986.1	hypothetical protein [Klebsiella phage UTI-K4]	513	-	100.00%	4.00E-111
210	QQM14318.1	hypothetical protein [Klebsiella phage vB_KpnM_17-11]	369	-	98.36%	5.00E-85
211	YP_009289579.1	hypothetical protein BI014_gp178 [Klebsiella phage PKO111]	207	-	98.53%	1.00E-38
212	ULA52450.1	rIII lysis inhibitor accessory [Enterobacter phage vB-EclM_KMB19]	249	-	79.27%	9.00E-40
213	ULA52451.1	capsid assembly chaperone [Enterobacter phage vB-EclM_KMB19]	324	-	75.47%	1.00E-52
214	QPB08810.1	tail fiber protein [Klebsiella phage Metamorpho]	309	-	97.06%	5.00E-67
215	AUV57563.1	hypothetical protein [Klebsiella phage KP1]	201	-	100.00%	4.00E-38
216	YP_009289575.1	deoxycytidylate deaminase [Klebsiella phage PKO111]	570	-	99.47%	2.00E-137
217	UGO49585.1	hypothetical protein MERCI_123 [Klebsiella phage vB_KaeM_Merci]	345	-	93.86%	3.00E-73
218	YP_009289573.1	hypothetical protein BI014_gp172 [Klebsiella phage PKO111]	237	-	98.72%	9.00E-50
219	QYC52921.1	hypothetical protein [Klebsiella phage	285	-	98.94%	9.00E-6

		vB_KpnM_TU02]				2
220	QBA85065.1	3'-phosphatase, 5'-polynucleotide kinase [Klebsiella phage vB_KpnM_GF]	894	-	100.00%	0.00E+00
221	UMM76998.1	hypothetical protein [Klebsiella phage UTI-K4]	213	-	97.14%	1.00E-42
222	YP_010098467.1	outer membrane lipoprotein [Klebsiella phage KP179]	288	-	97.89%	5.00E-63
223	QGF21640.1	spanin Rz [Klebsiella phage JIPh_Kp122]	354	-	98.29%	2.00E-77
224	QPB08801.1	Alc-like transcription inhibitor [Klebsiella phage Metamorpho]	510	-	98.22%	4.00E-112
225	QLF83100.1	RNA ligase [Klebsiella phage KpnM6E1]	1152	-	99.19%	0.00E+00
226	QQM14302.1	endonuclease [Klebsiella phage vB_KpnM_17-11]	417	-	98.55%	2.00E-95
227	QEG11614.1	ribonucleotide reductase of class Ia [Klebsiella phage KPN6]	1143	-	99.47%	0.00E+00
228	YP_009190801.1	ribonucleoside-diphosphate reductase subunit alpha [Klebsiella phage JD18]	2247	-	100.00%	0.00E+00
229	UCR74167.1	hypothetical protein [Klebsiella phage vB_KpnM_5N]	207	-	91.18%	2.00E-38
230	AUV57581.1	thymidylate synthase [Klebsiella phage KP1]	876	-	98.63%	0.00E+00
231	YP_009190805.1	hypothetical protein AU097_gp224 [Klebsiella phage JD18]	318	-	99.05%	3.00E-71
232	YP_009190806.1	dihydrofolate reductase [Klebsiella phage JD18]	600	-	97.99%	3.00E-143
233	YP_010098455.1	hypothetical protein KNU12_gp015 [Klebsiella phage KP179]	321	-	96.23%	3.00E-70
234	UOK17848.1	hypothetical protein KP1079_00242 [Klebsiella phage KP1079]	279	-	98.75%	2.00E-51
235	QEG11714.1	hypothetical protein KPN6_71 [Klebsiella phage KPN6]	279	-	98.91%	1.00E-58
236	YP_009190811.1	hypothetical protein AU097_gp230 [Klebsiella phage JD18]	222	-	98.63%	3.00E-45
237	UJP30156.1	hypothetical protein [Klebsiella phage Kpn6N]	258	-	98.82%	5.00E-54
238	QEG11081.1	single-stranded DNA-binding protein [Klebsiella phage KMI12]	918	-	99.66%	2.00E-174
239	UGO53394.1	homing endonuclease [Klebsiella phage vB_KaeM_Nispero]	663	-	99.09%	1.00E-163
240	QPB08784.1	DNA helicase loader [Klebsiella phage Metamorpho]	663	-	99.52%	3.00E-148
241	UMM77020.	transcriptional regulator [Klebsiella phage UTI-K4]	300	-	98.99%	1.00E-6

	1					4
242	UYE90783.1	double-stranded DNA binding protein [Klebsiella phage pKp20]	273	-	98.89%	9.00E-56
243	UMM76742.1	ribonuclease H [Klebsiella phage UTI-K1]	927	-	99.35%	0.00E+00
244	AUV57596.1	long tail fiber proximal subunit [Klebsiella phage KP1]	3846	+	98.52%	0.00E+00
245	YP_010089575.1	tail connector protein [Klebsiella phage KPV15]	1137	+	97.88%	0.00E+00
246	UMM76745.1	tail fibers protein [Klebsiella phage UTI-K1]	693	+	97.83%	3.00E-138
247	UGO48321.1	hypothetical protein SHINKOU_259 [Klebsiella phage vB_KaeM_Shinkou]	4173	+	79.11%	0.00E+00
248	YP_009190822.1	hypothetical protein AU097_gp241 [Klebsiella phage JD18]	405	+	99.25%	5.00E-84
249	YP_009289543.1	holin [Klebsiella phage PKO111]	657	+	98.17%	6.00E-141
250	UJP30168.1	anti-sigma factor [Klebsiella phage Kpn6N]	273	-	98.89%	6.00E-57
251	QEG11765.1	hypothetical protein KPN6_86 [Klebsiella phage KPN6]	267	-	98.86%	1.00E-57
252	AUV57604.1	hypothetical protein [Klebsiella phage KP1]	327	-	100.00%	1.00E-72
253	QYC52955.1	hypothetical protein [Klebsiella phage vB_KpnM_TU02]	279	-	98.91%	1.00E-61
254	YP_009190828.1	hypothetical protein AU097_gp247 [Klebsiella phage JD18]	126	-	100.00%	4.00E-19
255	YP_010098694.1	anti-restriction nuclease [Klebsiella phage KP179]	303	-	98.00%	2.00E-65
256	UNY41062.1	anti-restriction nuclease [Klebsiella phage KP182]	426	-	99.29%	2.00E-101
257	UGO53412.1	anti-restriction nuclease [Klebsiella phage vB_KaeM_Nispero]	342	-	97.35%	3.00E-76
258	QEG11407.1	transcriptional regulator of middle promoters [Klebsiella phage KMI13]	645	-	97.66%	8.00E-149
259	UNY41346.1	hypothetical protein [Klebsiella phage KP185]	264	-	98.85%	5.00E-56
260	YP_009190835.1	DNA topoisomerase [Klebsiella phage JD18]	1389	-	100.00%	0.00E+00
261	YP_010096245.1	hypothetical protein KNT92_gp141 [Klebsiella phage Mineola]	156	-	100.00%	3.00E-27
262	AUV57616.1	hypothetical protein [Klebsiella phage KP1]	456	-	100.00%	5.00E-108
263	YP_0100962	hypothetical protein KNT92_gp139 [Klebsiella phage	192	-	98.41%	1.00E-3

	47.1	Mineola]				5
264	YP_0091908 40.1	hypothetical protein AU097_gp259 [Klebsiella phage JD18]	117	-	100.00%	7.00E-1 6
265	YP_0091908 41.1	hypothetical protein AU097_gp260 [Klebsiella phage JD18]	240	-	98.73%	3.00E-4 9
266	YP_0091908 42.1	hypothetical protein AU097_gp261 [Klebsiella phage JD18]	168	-	100.00%	6.00E-3 1

Table S2. CDS function prediction of phage PST-H1.

C DS	Hit_name	Hit_description	Length (aa)	Stra nd	Identity (%)	Value
1	QIG57273.1	tail fiber protein [Salmonella phage vB_SpuP_Spp11]	2559	-	99.53%	0.00E+ 00
2	YP_0088596 51.1	hypothetical protein SPSV3_gp29 [Salmonella phage SETP3]	366	-	100.00%	2.00E-8 5
3	ALN97486.1	hypothetical protein [Salmonella phage fSE1C]	516	-	99.42%	2.00E-1 21
4	UJP30006.1	hypothetical protein [Salmonella phage CKT1]	444	-	100.00%	3.00E-1 05
5	UJP30007.1	tail tape measure [Salmonella phage CKT1]	2334	-	99.61%	0.00E+ 00
6	UJP30008.1	hypothetical protein [Salmonella phage CKT1]	360	-	100.00%	4.00E-8 3
7	UHM94159. 1	hypothetical protein [Salmonella phage vB_SenS_TUMS_E19]	417	-	98.55%	4.00E-9 6
8	AXQ70283.1	hypothetical protein vst10_25 [Salmonella virus VSt10]	180	+	71.43%	5.00E-1 9
9	QQK87850.1	hypothetical protein [Salmonella phage SLMP1]	1140	+	93.88%	0.00E+ 00
10	DAL23253.1	TPA_asm: MAG TPA_asm: homing endonuclease [Siphoviridae sp.]	474	+	100.00%	2.00E-7 6
11	UKM96712.1	hypothetical protein PBSE191_38 [Salmonella phage PBSE191]	231	+	98.68%	3.00E-4 7
12	UGC97872.1	DNA-binding protein [Salmonella phage LP31]	672	+	98.21%	4.00E-1 61
13	AXC39501.1	hypothetical protein [Salmonella phage S100]	1170	-	99.49%	0.00E+ 00
14	APU03010.1	hypothetical protein LPSE_00055 [Salmonella phage LPSE1]	420	-	98.56%	5.00E-9 6
15	QDH44714.1	hypothetical protein [Salmonella phage SF4]	396	-	98.47%	1.00E-7 2
16	QIG57288.1	hypothetical protein [Salmonella phage vB_SpuP_Spp11]	360	-	96.64%	2.00E-7 6

17	UJP30019.1	hypothetical protein [Salmonella phage CKT1]	606	-	97.51%	9.00E-130
18	QNI20459.1	hypothetical protein SHWT1_30 [Salmonella phage SHWT1]	510	-	99.41%	8.00E-119
19	YP_001110840.1	hypothetical protein SPSV3_gp13 [Salmonella phage SETP3]	189	-	96.77%	9.00E-35
20	APM00275.1	neck whiskers protein [Salmonella phage STP03]	351	-	97.41%	5.00E-74
21	UJP30024.1	capsid and scaffold [Salmonella phage CKT1]	288	-	96.84%	2.00E-39
22	UJP30025.1	major capsid protein [Salmonella phage CKT1]	1050	-	99.40%	0.00E+00
23	AFO70806.1	hypothetical protein [Salmonella phage ST4]	702	-	99.14%	3.00E-127
24	ASZ77968.1	hypothetical protein [Salmonella phage ST3]	390	-	98.45%	6.00E-87
25	ASZ77969.1	hypothetical protein [Salmonella phage ST3]	120	-	89.74%	3.00E-16
26	AXC39811.1	neck whiskers protein [Salmonella phage S106]	459	-	97.69%	3.00E-85
27	QNJ54499.1	hypothetical protein [Salmonella phage UPWr_S5]	1860	-	92.73%	0.00E+00
28	AXC39483.1	hypothetical protein [Salmonella phage S100]	654	+	93.55%	2.00E-152
29	QVQ56298.1	62 kDa structural protein [Salmonella phage JD01]	1476	-	98.98%	0.00E+00
30	QIO03746.1	terminase [Salmonella phage skrot]	1272	-	99.53%	0.00E+00
31	UJP30034.1	hypothetical protein [Salmonella phage CKT1]	507	-	92.26%	7.00E-98
32	UJP30036.1	hypothetical protein [Salmonella phage CKT1]	180	-	55.88%	1.00E-04
33	UMW88037.1	hypothetical protein [Escherichia phage UTI-CM001]	237	-	98.72%	5.00E-49
34	QQV88101.1	hypothetical protein [Escherichia phage phiWAO78-1]	222	-	90.74%	4.00E-29
35	QYC53070.1	hypothetical protein [Salmonella phage vB_SalS_TU03]	291	-	79.17%	2.00E-14
36	QYC53069.1	hypothetical protein [Salmonella phage vB_SalS_TU03]	177	-	66.67%	7.00E-19
37	QMS41844.1	hypothetical protein S55_GM000032 [Salmonella phage S55]	156	-	96.08%	2.00E-27
38	YP_009620149.1	hypothetical protein FDJ09_gp69 [Escherichia phage VB_EcoS-Golestan]	150	-	95.92%	4.00E-24

39	QZI78493.1	lysin [Escherichia phage vB_EcoS-22664BS2]	492	-	89.44%	2.00E-67
40	YP_008767059.1	putative class I holin [Salmonella phage SETP13]	291	-	96.88%	7.00E-50
41	YP_001110821.1	hypothetical protein SPSV3_gp53 [Salmonella phage SETP3]	282	-	100.00%	1.00E-60
42	QQK87823.1	hypothetical protein [Salmonella phage SLMP1]	366	-	96.69%	3.00E-62
43	APM00302.1	hypothetical protein STP03_048 [Salmonella phage STP03]	204	-	92.54%	3.00E-40
44	UJP30047.1	hypothetical protein [Salmonella phage CKT1]	402	-	100.00%	4.00E-88
45	QMS41853.1	hypothetical protein S55_GM000041 [Salmonella phage S55]	165	-	100.00%	4.00E-32
46	UJP29985.1	hypothetical protein [Salmonella phage CKT1]	117	-	100.00%	4.00E-18
47	QNI20430.1	hypothetical protein SHWT1_1 [Salmonella phage SHWT1]	171	+	98.21%	1.00E-32
48	AFO70782.1	hypothetical protein [Salmonella phage ST4]	234	+	97.40%	3.00E-46
49	QQK87829.1	replicative DNA helicase [Salmonella phage SLMP1]	2187	+	98.76%	0.00E+00
50	AXC39658.1	hypothetical protein [Salmonella phage S102]	219	-	98.61%	2.00E-44
51	UMO77768.1	13.88 kDa late protein [Salmonella phage F118P13]	513	+	97.65%	4.00E-72
52	UJP29992.1	hypothetical protein [Salmonella phage CKT1]	1437	+	96.83%	0.00E+00
53	UJP29993.1	hypothetical protein [Salmonella phage CKT1]	627	+	98.94%	1.00E-134
54	UJP29994.1	DNA polymerase I [Salmonella phage CKT1]	2202	+	99.73%	0.00E+00
55	AXC39521.1	hypothetical protein [Salmonella phage S100]	132	+	88.37%	1.00E-16
56	AWY03026.1	hypothetical protein [Salmonella phage vB_SpuS_Sp4]	288	+	93.68%	2.00E-60
57	YP_007010488.1	hypothetical protein F491_gp35 [Salmonella phage vB_SenS-Ent1]	192	+	100.00%	1.00E-36
58	UJP29999.1	hypothetical protein [Salmonella phage CKT1]	684	+	96.04%	2.00E-163
59	UJP30000.1	DNA helicase [Salmonella phage CKT1]	1341	+	100.00%	0.00E+00
60	UIW13727.1	hypothetical protein [Salmonella phage vB_SalS-S10]	183	+	98.33%	1.00E-20

61	QNI20443.1	tail fibers protein [Salmonella phage SHWT1]	735	-	96.70%	5.00E-125
